# Green physical activity for leisure connects perceived residential greenspace and mental well-being

**DOI:** 10.3389/fpubh.2023.1254185

**Published:** 2023-10-04

**Authors:** Hansen Li, Yun Li, Zhenhuan Wang, Guodong Zhang

**Affiliations:** ^1^College of Physical Education, Southwest University, Chongqing, China; ^2^Institute for Health and Sport (iHeS), Victoria University, Melbourne, VIC, Australia

**Keywords:** urban greening, neighborhood, greenery, recreation, model

## Abstract

Physical activity serves as a pivotal mediator in previous theoretical frameworks that link greenspace and human health. However, it remains unclear whether the domain of physical activity within and around greenspaces can alter the pathway. The present study recruited 668 participants online and examined a conceptual framework that explores the associations between residential greenspace and mental well-being, with a particular focus on the mediation effect of green physical activity (physical activity undertaken in and around greenspaces). Moreover, socio-demographic characteristics, including gender, age, household income, education status, marital status, and student status, were controlled for during the examination. The investigated green physical activities included leisure activities, transportation walking, and transportation cycling, and they were measured by a pre-established questionnaire. Meanwhile, mental well-being was measured by the WHO-5 well-being index, and residential greenspace was indicated by self-reported perceived greenspace and mean Normalized Difference Vegetation Index (NDVI) values within 500 meters (m) of residential radius. We found that both perceived greenspace (*B* = 1.852, *p* < 0.001) and NDVI _500 m_ (*B* = 3.230, *p* = 0.038) were positively associated with mental well-being. However, only perceived greenspace, not NDVI 500 m, exhibited positive associations with the three green physical activity items. Furthermore, only green physical activity for leisure (*B* = 0.223, *p* < 0.001), not for transportation (*p* > 0.05), mediated the relationship between perceived greenspace and mental well-being. Our findings reinforce previous studies on “greenspace-health” frameworks and underline the importance of leisure physical activity in promoting mental well-being.

## 1. Introduction

Greenspace plays a pivotal role in the promotion of public health, such as reducing negative emotions, promoting postoperative rehabilitation, and preventing infectious diseases (1). Among different greenspaces, residential greenspace is particularly beneficial due to its long-lasting impacts on residents (2, 3). Existing literature has demonstrated that higher levels of residential greenspace are associated with improved mental health and well-being (4, 5). To date, numerous theories and frameworks have been developed to investigate the mechanisms that underlie these positive associations. For example, lower psychological stress and greater social contact are potential mediators between greenspace and health/well-being. Notably, physical activity is often mentioned as a key mediator (6–8). This mediatory role of physical activity is primarily based on the assumption that greenspace may reduce disturbances such as air pollution, noise, and heat, consequently creating a comfortable environment for physical activity (6, 7, 9). Moreover, residential greenspace may also foster an emotional attachment to nature and attract people to interact with nature, thereby increasing their physical activity levels (2).

Physical activity in natural settings, such as greenspace, is sometimes defined as green physical activity (10, 11). This concept has attracted significant research attention due to its potential benefits derived from nature exposure and physical activity (12). Although many studies have explored the relationship between greenspaces and physical activity, few have confirmed whether the observed physical activities actually took place within the greenspaces. Consequently, their findings may not effectively support the existing assumption that greenspaces promote physical activity by providing comfortable environments (6). For example, some physical activities may occur indoors, such as in gyms or urban centers, particularly considering the growing reliance on urban facilities among urban residents (13, 14). Furthermore, while recent studies have identified positive associations between residential greenspace and green physical activity, there are also studies that report non-significant associations between the two (15, 16). This discrepancy could be attributed to the variations in modes/types of physical activity (17) and the measures of greenspace (18). These uncertainties leave the pathway from residential greenspace to green physical activity unclear.

**Figure 1:**
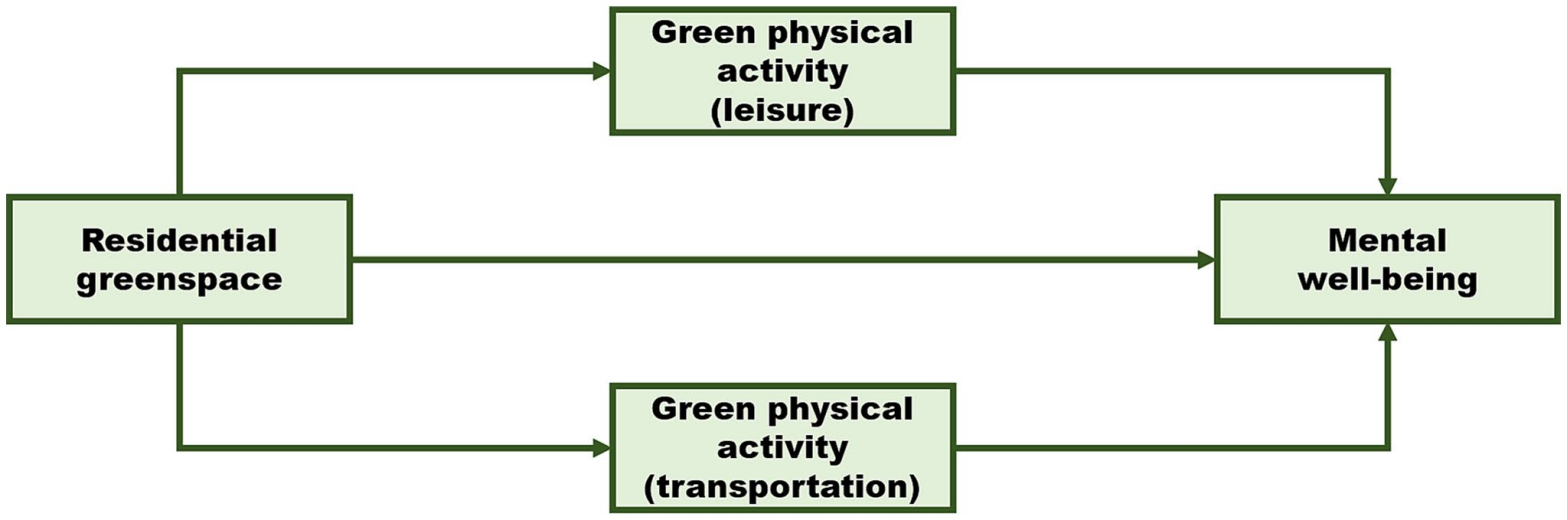
The conceptual framework.

Typically, physical activity can be categorized by various domains, including “leisure,” “transportation,” “occupation,” and “household” physical activity (19, 20). Greenspaces are public open areas that often contain recreational facilities or are strategically situated alongside roads to mitigate environmental disturbances, making them particularly conducive to leisure and transportation-related physical activities. Thus, the present study aimed to investigate the association between residential greenspace and mental well-being through these specific green physical activities. To visualize our research objectives, we developed a conceptual model as follows (Figure 1). Our hypotheses were:

*H1:* Different green physical activities are positively associated with mental well-being.

*H2:* Residential greenspace is positively associated with green physical activities and mental well-being.

*H3:* Different green physical activities mediate the relationship between residential greenspace and mental well-being.

## 2. Materials and methods

### 2.1. Study design and participants

We conducted an online cross-sectional survey from April 25–30, 2023. We aimed to recruit urban residents as our participants. Due to our limited research sources, we distributed recruiting messages to volunteer chat groups (e.g., survey volunteer and community volunteer groups) through QQ and WeChat platforms. We also used the snowball method to encourage the initially recruited participants to call for participation through their social circles. The study’s topic was described as an investigation of residential urban greening and physical activity. Details of the research questions were not disclosed. We offered compensation of 5 renminbi (RMB) for participation. It is noteworthy that one RMB was roughly equivalent to 0.14 US dollars as of August 2023. Participants were required to use WeChat accounts that linked their personal legal IDs so as to ensure that they were real people. Their device, WeChat ID, and IP address were monitored to avoid repeated participation. Inclusion criteria were: (1) having a fixed residence in cities; (2) living in the fixed residence during the recent month; and (3) being at home while completing the questionnaire. Exclusion criteria were: (1) no informed consent was provided; (2) questionnaires with unfinished items; (3) individuals who failed verification tests (to confirm that the questionnaire was carefully completed by people instead of a machine or program or random filling); and (4) at places other than home (verified through questions in the survey). Considering the potential ethical issues in GPS-based studies (21), we collected momentary data (not continuous or historical data) through a third-party platform (GaoDe AutoNavi Holdings Ltd.) that is licensed and supervised by the Chinese government. The geo-information was uploaded manually by the participants (we were not able to access their smartphones). We explained our use of location information, including purpose, accuracy, and the form of the information, before the survey began. Written consent was obtained from all individuals before the investigation (those who did not submit consent material were not allowed to participate). Furthermore, our survey is conducted entirely anonymously to avoid the invasion of privacy. This study was approved and supervised by the Ethics Review Board of Southwest University.

### 2.2. Instruments and measurements

#### 2.2.1. Greenspace assessment

We considered both measured and perceived greenspace for indicating the overall amount of greenspace in residential neighborhoods.

##### 2.2.1.1. Measured greenspace

We used a locating service empowered by GaoDe AutoNavi Holdings Ltd. to capture the coordinates of our participants via the GPS functions in their smartphones. We used the Normalized Difference Vegetation Index (NDVI) to reflect measured greenspace, which is a useful measure of neighborhood greenness that has practical advantages and may aid in replication and comparability across studies (22, 23). We used Google Earth Engine (GEE) to obtain the maximum annual NDVI of China (for the year 2022) based on a MODIS product from NASA (code: MOD13Q1), which has a 250×250 meter (m) spatial resolution. Thereafter, to avoid the NDVI observation of water masking the existence of vegetation, we followed the recommendations of others by removing negative values before analysis (5, 6). We referred to previous studies and established buffers with a 500 m radius around participants’ residences (4, 24). The mean NDVI value extracted from the buffers was regarded as the overall greenness in residential neighborhoods.

##### 2.2.1.2. Perceived greenspace

We asked the participants to estimate the overall amount of greenspace in their residential neighborhood (25–28). The statement was “Please rate the greenspace level (amount) in your neighborhood. For example, are there many trees and grassland?” The response was captured by a 10-point NRS scale, where 1 = none and 10 = extremely sufficient. Previous studies have found that data from such a measure on perceived greenspace is associated with mental health in a similar population (25, 26), which suggests the measure has relevance to mental health.

#### 2.2.2. Green physical activity

For leisure green physical activity, we modified the items by Troped et al. (29) to match the green physical activity scenarios. We used “How many days per week in the past month have you engaged in leisure physical activity or exercise (such as walking and aerobics) in a greenspace (e.g., with trees and grassland)?” to measure the frequency of leisure green physical activity. The results were measured using an 8-point Likert scale (where 1 point indicates “never” and 8 points indicates “almost every day”). Meanwhile, we used “How long did each bout of the above-mentioned activities last on average?” to measure the duration of each bout of leisure green physical activity. The results were measured using a 10-point Likert scale (where 1 point indicates “0–10 min” and 10 points indicates “90 min or more”). The product of the scores for both options was considered the total score of leisure green physical activity. For transportation green physical activity, we used two questions by Troped et al. (29) to measure walking and cycling-related green physical activity. According to Troped et al. (29), the questions are from the MOSPA survey on walking and bicycling. The question about walking was “On average, how long did you walk in a greenspace (e.g., through a park or community trail) for transportation purposes per day in the past month?” The question about cycling was “On average, how long did you cycle in a greenspace (e.g., through a park or community trail) for transportation purposes per day in the past month?” The results of both questions were measured using a 10-point Likert scale (1 point indicates “0–10 min” and 10 points indicates “90 min or more”).

#### 2.2.3. Mental well-being

The WHO-5 well-being index was used to measure mental well-being, as suggested by previous studies (5, 30). This scale consists of seven items, such as “I have felt calm and relaxed” and “I woke up feeling fresh and rested.” Answers were obtained on a 5-point Likert-type scale (0 = never, 4 = every time). To better associate mental well-being with our other variables (e.g., green physical activity), we used “over the last month” as the time frame for these questions. We used a Chinese version of WHO-5, and it showed good internal consistency in the current study (Cronbach *α* > 0.9). The summary score of WHO-5 was used to represent mental well-being in the subsequent analyses.

#### 2.2.4. Control variables

We included sociodemographic factors as control variables to adjust for the associations in our framework. Gender, age, and marital status may influence individuals’ likelihood of visiting greenspaces and their mental well-being (31, 32). Household income and education status are critical indicators of an individual’s socioeconomic status (SES) (33), which may affect both the likelihood of residing in areas with abundant greenery and mental health outcomes (34). Similarly, student status, which is associated with campus life, can significantly impact individuals’ residential environment (e.g., campus greening) as well as their behavior and health, and was therefore included as a control variable as well. These sociodemographic factors were measured as follows:

Gender (0 = male and 1 = female), age (coded as: 1, ≤15; 2, 15–25; 3, 26–35; 4, 36–45; 5, 46–55; 6, 56–65; 7, 66–75; 8, ≥75 years), education status (coded as: 1, lower than undergraduate; 2, undergraduate; 3, higher than undergraduate), household income (coded as: 1, 0–5,000 RMB; 2, 5,001–10,000 RMB; 3, 10,001–15,000 RMB; 4, 15,001–20,000 RMB; 5, 20,001–25,000 RMB; 6,>25,000 RMB monthly), and student status (0 = yes and 1 = no), marital status (0 = yes and 1 = no).

### 2.3. Analysis

#### 2.3.1. Correlation

Given the nonnormal data, Spearman’s rank-order correlation was used to probe for general correlations between two continuous variables of interest.

#### 2.3.2. Structural equations modeling

Based on our employed measures, we developed a framework with three green physical activity mediators. Considering the potential interrelationships among the three types of green physical activities, we established covariance links to account for co-variation. The specific conceptual model was demonstrated in Supplementary Figure S1.

Structural equation modeling (SEM) was employed to investigate the hypothesized associations and mediation effect. According to MacCallum et al. (35), the sample size for SEM should be ten times the number of model parameters or more. Based on the parameters (*n* = 42) required to estimate in the framework, our sample size of 668 was adequate.

Variance Inflation Factor (VIF) values smaller than 5.0 were considered evidence of the absence of multicollinearity. Based on this rule, no multicollinearity was observed among the independent variables (VIF < 3.0) (36).

The analysis was conducted using the Maximum Likelihood (ML) estimator. To cope with the nonnormality, we used the bias-corrected bootstrap method (37) with 10,000 replications to generate corresponding standard errors and confidence intervals for all paths (38–40). Meanwhile, given that the χ^2^ test is strongly affected by nonnormality (41–43), we turned to the Bollene-Stine statistic to check the overall model fit (44–46), where *p*_Bollene-Stine_ > 0.05 is a sign of acceptable model fit. Additionally, common model fitting indices were also reported. Their titles and criteria were: Standardized Root Mean Square Residual (SRMR) < 0.08; Tucker-Lewis Index (TLI) > 0.95; Comparative Fit Index (CFI) > 0.95; Root Mean Square Error of Approximation (RMSEA) < 0.05 (47).

An indirect effect (i.e., a product of coefficients for the constituent links) that significantly exceeded zero was evidence of mediation (48, 49). The total effects in the model were not interpreted as causality, but as the total associations realized through direct and indirect pathways.

All the statistical analyses were conducted in SPSS 26.0 and AMOS 26.0 software.

## 3. Results

### 3.1. Participants characteristics

Our participants were mainly male, aged between 26 and 35 years, and having a monthly household income of 50,001 to 10,000 RMB (Supplementary Table S1). Around 60% of participants were unmarried, 47.9% were students, and 63.8% declared to have experienced undergraduate education.

### 3.2. Correlations between variables of interest

We found that NDVI 500 m was negatively correlated with green cycling for transportation (*ρ* = −0.090, *p* = 0.020). Perceived greenspace was positively correlated with green physical activity for leisure (*ρ* = 0.282, *p* < 0.001), green walking for transportation (*ρ* = 0.114, *p* = 0.003), and mental well-being (*ρ* = 0.534, *p* < 0.001) (Supplementary Figure S2). Mental well-being was positively correlated with green physical activity for leisure (*ρ* = 0.338, *p* < 0.001) and green walking for transportation (*ρ* = 0.133, *p* = 0.001).

### 3.3. Pathway and mediation analysis

#### 3.3.1. Model modification

Since only perceived greenspace was found to positively associate with both green activities and mental well-being, we loaded the conceptual model with perceived greenspace for assessing the hypothesized mediation effect. The initial model showed poor model fits (*p*_Bollene-Stine_ = 0.007; SRMR = 0.016; CFI = 0.994; TLI = 0.886; RMSEA = 0.068 [90%CI: 0.031 to 0.109]). Therefore, we followed the modification indicator and built a covariance link between perceived greenspace and age. This modification is supported by previous studies suggesting that age is associated with changes in greenspace use and perception (32, 50). After the adjustment, the model showed acceptable model fits (*p*_Bollene-Stine_ = 0.214; SRMR = 0.009; CFI = 0.999; TLI = 0.981; RMSEA = 0.027 [90%CI: 0.000 to 0.086]) and was therefore selected as the final model. After that, we loaded the final model with NDVI 500 m and also obtained acceptable model fits (*p*_Bollene-Stine_ = 0.250; SRMR = 0.008; CFI = 0.999; TLI = 0.986; RMSEA = 0.022 [90%CI: 0.000 to 0.083]).

#### 3.3.2. The associations between green physical activities and mental well-being

**Figure 2:**
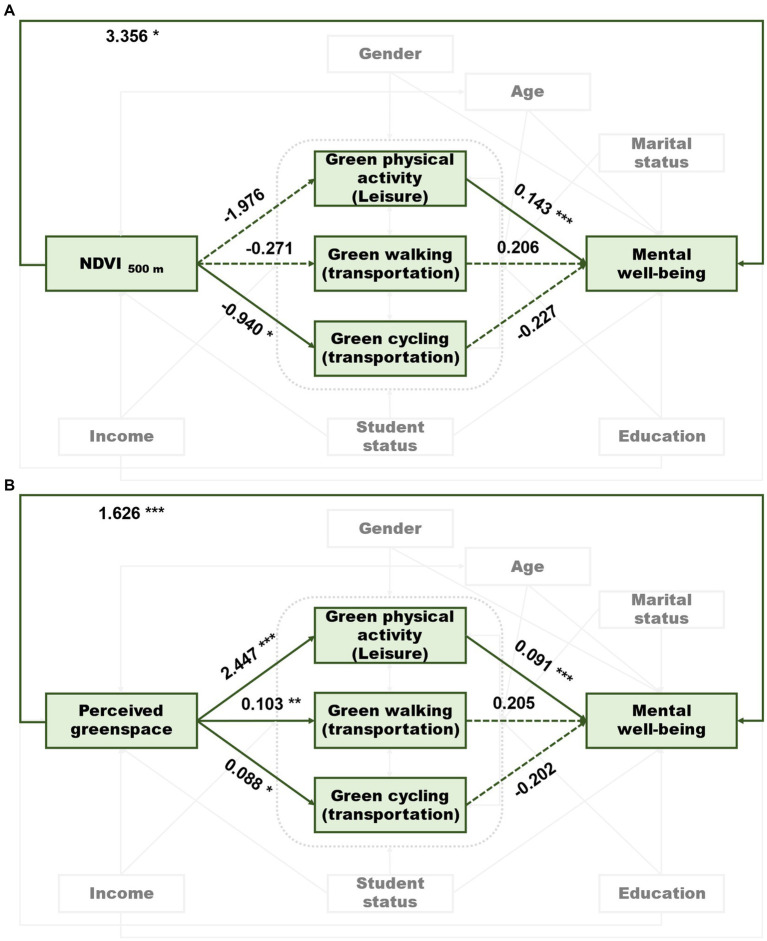
The final models showing the pathways from NDVI 500 m **(A)** or perceived greenspace **(B)** to mental well-being. Solid lines indicate significant pathways and dotted lines indicate non-significant pathways. Numbers are unstandardized regression coefficients.

Only green leisure physical activity was found to positively associate with mental well-being when the three green physical activity items were simultaneously loaded as predictors in the two models (loaded with NDVI 500 m or perceived greenspace) (Figure 2).

#### 3.3.3. The associations of greenspace indicators to green physical activities and mental well-being

According to the model loaded with NDVI 500 m, NDVI 500 m was negatively associated with green cycling for transportation (*p* = 0.014) but was positively associated with mental well-being (*p* = 0.038). In the other model loaded with perceived greenspace, perceived greenspace was positively associated with the three green physical activity items and also mental well-being (Table 1).

**Table 1:** Total effects of greenspace indicators on green physical activities and mental wellbeing.

Greenspace indicator	Green physical activities	B (95% CI)	*p*
NDVI_500 m_	Green PA (leisure)	−1.976 (−8.835, 4.519)	0.534
	Green walking (transportation)	−0.271 (−0.949, 0.403)	0.426
	Green cycling (transportation)	−0.940 (−1.680, −0.206)	0.014
	Mental well-being	3.230 (0.176, 6.189)	0.038
Perceived greenspace	Green PA (leisure)	2.447 (0.033, 0.173)	<0.001
	Green walking (transportation)	0.103 (1.787, 3.133)	0.006
	Green cycling (transportation)	0.088 (0.009, 0.166)	0.030
	Mental well-being	1.852 (1.590, 2.109)	<0.001

B, regression coefficient; CI, confident interval; PA, physical activity.

#### 3.3.4. The mediatory role of green physical activities between greenspace and mental well-being

As shown in Table 2, green leisure physical activity was the only mediator to link perceived greenspace and mental well-being (*p* < 0.05). This pathway contributed to 12.04% of the total effect of perceived greenspace on mental well-being.

**Table 2:** Indirect pathways from greenspace to mental well-being.

Pathway	B (95% CI)	*p*
Perceived greenspace → Green PA (leisure) → Mental well-being	0.223 (0.136, 0.324)	<0.001
Perceived greenspace → Green walking (transportation) → Mental well-being	0.021 (−0.013, 0.078)	0.199
Perceived greenspace → Green cycling (transportation) → Mental well-being	−0.018 (−0.074, 0.007)	0.165
NDVI_500 m_ → Green PA (leisure) → Mental well-being	−0.283 (−1.304, 0.634)	0.521
NDVI_500 m_ → Green walking (transportation) → Mental well-being	−0.056 (−0.454, 0.066)	0.279
NDVI_500 m_ → Green cycling (transportation) → Mental well-being	0.213 (−0.055, 0.746)	0.117

B, regression coefficient; CI, confident interval; PA, physical activity.

## 4. Discussion

The primary objective of this study was to investigate the mediating effects of green physical activities on the relationship between residential greenspace and mental well-being. Our findings revealed positive associations of both measured and perceived greenspace with mental well-being. Moreover, we identified leisure green physical activity as a significant mediator in the pathway from perceived greenspace to mental well-being. These results not only support previous studies on the association between greenspace and health (5, 6) but also emphasize the special role of leisure physical activity in this context.

### 4.1. Green physical activities and mental well-being

Green physical activities, also referred to as green exercise in many studies, have been anticipated to combine the health benefits of physical activity and nature exposure. However, previous research has often overlooked the distinctions among green physical activities within various domains. In this study, we found that while green physical activity for leisure and transportation (specifically walking) exhibited positive correlations with mental well-being, only green physical activity for leisure, but not transportation, demonstrated a significant positive association with mental well-being in our model. This finding partially supports our first hypothesis (H1). Nevertheless, this result is inconsistent with some studies where mental well-being is positively associated with active transportation such as walking and cycling (51–53).

A possible explanation for this discrepancy lies in the fact that previous studies often focused on a single domain of physical activity without adequately controlling for other physical activities. For instance, individuals who have cultivated an active lifestyle through leisure physical activities may also prefer engaging in physical activity during transportation. In other words, leisure physical activity could confound the association between transportation physical activity and mental well-being. Therefore, it may be essential to consider the physical activities of different domains to reveal the true associations.

The observed disparity between green physical activity for leisure and transportation is understudied yet. However, it may be explained by the different tasks involved in each activity. Specifically, previous studies have suggested that the mental health benefits of green physical activity may be related to the restorative experiences during nature exposure (54–56), and the restorative experiences can be affected by the task within the activity, with those tasks requiring more directed attention leading to impaired benefits (57). Transportation green physical activity often entails a stressful or attention-consuming task (e.g., reaching the workplace or school on time), which may lead to lower mental health benefits. By comparison, leisure greenspace activity is usually carried out for recreation, which may demand less attention or other mental resources, thereby offering greater benefits.

### 4.2. Residential greenspace, green physical activities, and mental well-being

We observed a positive association between perceived greenspace and the three green physical activity items. In contrast, the association between measured greenspace (NDVI 500 m) and green cycling for transportation was negative. These findings only partially support our second hypothesis. While seemingly contradictory, these outcomes are not entirely unexpected. This is because NDVI only reflects the general “amount” of greenspace rather than its quality or accessibility (5). In China, urban greenspace development lags behind that of Western developed countries, resulting in many greenspaces being undeveloped areas rather than well-maintained urban green facilities. This characteristic makes them less likely to be used for leisure and transportation physical activity.

Notably, In our correlation analysis, perceived greenspace was not significantly correlated to measured greenspace, which is inconsistent with some studies (58) but somewhat supports the aforementioned assumption. These greenspaces may lack designated areas or pathways for activities, or they may be obstructed from view by walls or other obstacles. Consequently, participants are unlikely to perceive and use those greenspaces. Even worse, those untapped and unavailable greenspaces may even hinder green physical activities by occupying the land that could otherwise be used for available greenspace construction. As mentioned in a previous study (59), such inaccessible, non-walkable, or even invisible greenspaces may not even exist in the mental maps of residents. In contrast, perceived greenspace may be closely related to usable greenspaces, thus effectively predicting green physical activities. These findings partly support the notion that subjective variables (e.g., perceived greenspace) are stronger predictors of greenspace visitation (18, 60).

Regarding mental well-being, both measured and perceived greenspace exhibited positive associations with mental well-being. This finding supports our second hypothesis and aligns with relevant studies (5, 25). The positive association between perceived greenspace and mental well-being can be explained from many perspectives. On one hand, the mediation effects of green physical activity (as we identified) partially explained the association. On the other hand, extra pathways that are beyond our research scope may also contribute to this association. For example, regardless of accessibility and utilization, residential greenspaces may buffer environmental disturbances such as noise, air pollution, and urban heat (6, 7), and they may also improve emotional outcomes through visual contact through windows (30, 61, 62). These unmeasured pathways may collectively connect residential greenspace and mental well-being.

### 4.3. The mediatory role of green physical activity

In this study, we discovered that only leisure green physical activity mediated the association between residential greenspace and mental well-being. This finding partially supports our third hypothesis and offers a potential mechanism to comprehend the benefits of residential greenspace. As introduced earlier, while some studies have demonstrated the mediating role of physical activity between greenspace and mental health, few have investigated the specific locations where the physical activity took place (6). This issue/limitation may explain why some studies have reported weak mediation effects of physical activity (63). In response to this gap, our findings provide evidence to support the existing framework outlining how greenspace contributes to human health (6).

Furthermore, our findings highlight that green physical activity for leisure, rather than for transportation, is pivotal for this association. This finding aligns with previous studies. For example, a study indicated that cycling for commuting purposes did not mediate the relationship between greenspace and self-reported health in the Netherlands (64). Another study showed that increased levels of walking for recreational purposes explained the relationship between perceived greenspace and physical health (65). The two studies collectively imply the influence of the domain of physical activity on health benefits, although they did not investigate the places where physical activity occurred either.

Generally, our finding is unsurprising because leisure activity is known to promote mental well-being (66, 67), and this benefit may come from the fact that leisure activity provides an escape from routines and daily lives, and offers positive experiences, such as fascination with nature, reflection, and mental relaxation (68).

### 4.4. Limitations

In China, the age group below 15 years constitutes 1.75% of the total population, while those aged 65 years and older make up 14.2%. However, our participants were primarily aged between 15 and 35 years, which deviates from China’s general age structure. Considering that older Chinese residents tend to use greenspaces more often (69, 70), it is possible that we have underestimated greenspace utilization and its impact on mental well-being.

It is worth noting that our participant recruitment was limited to smartphone users in order to utilize the GPS function of smartphones. However, this approach introduces the potential for informant bias, as these participants may exhibit a stronger affinity towards electronic devices and modern technologies. Consequently, they might engage in fewer outdoor activities compared to individuals who infrequently or do not use smartphones.

The question items we used, including those for assessing perceived greenspace and green physical activity, are not from verified instruments. In fact, there is a lack of well-studied instruments for the two variables yet. As a result, our study might have been affected by high levels of measurement errors and common method bias. This flaw warrants future research to develop suitable measures and re-examine our findings.

Although the mediation analysis implies causal relationships between variables, it’s crucial to acknowledge that we adopted a cross-sectional design to capture data. Therefore, causality cannot be confirmed. Subsequent studies may consider employing longitudinal designs or instrumental variables to further examine causality.

## 5. Conclusion

This study aimed to investigate the relationship between residential greenspace and mental well-being. Additionally, it also explored the mediating pathways involving green physical activities for leisure or transportation. Our results revealed that both perceived and measured greenspace were positively associated with mental well-being. Furthermore, green physical activity for leisure emerged as a significant predictor of mental well-being and acted as a mediator in the association between residential greenspace and mental well-being. These findings provide location-specific evidence to support the existing framework or theory of “greenspace-physical activity-health” and emphasize the importance of leisure physical activity.

## Data availability statement

The raw data supporting the conclusions of this article will be made available by the authors, without undue reservation.

## Ethics statement

The studies involving humans were approved by the Ethics Review Board of Southwest University. The studies were conducted in accordance with the local legislation and institutional requirements. The participants provided their written informed consent to participate in this study. Written informed consent was obtained from the individual(s) for the publication of any potentially identifiable images or data included in this article.

## Author contributions

HL: Data curation, Formal analysis, Investigation, Writing – original draft, Writing – review & editing. YL: Funding acquisition, Supervision, Writing – review & editing. ZW: Resources, Writing – review & editing. GZ: Methodology, Project administration, Supervision, Writing – review & editing.

## Funding

This research is funded by the Transverse Project (No. 2308017) of Southwest University.
