# Supplementary material for: Green physical activity for leisure connects perceived residential greenspace and mental well-being
Source: Front Public Health. 2023 Oct 4;11:1254185. doi: 10.3389/fpubh.2023.1254185 (PMC10585364; doi:10.3389/fpubh.2023.1254185)
Supplement: Supplementary file 1 [file Data_Sheet_1.docx]

Supplementary Material


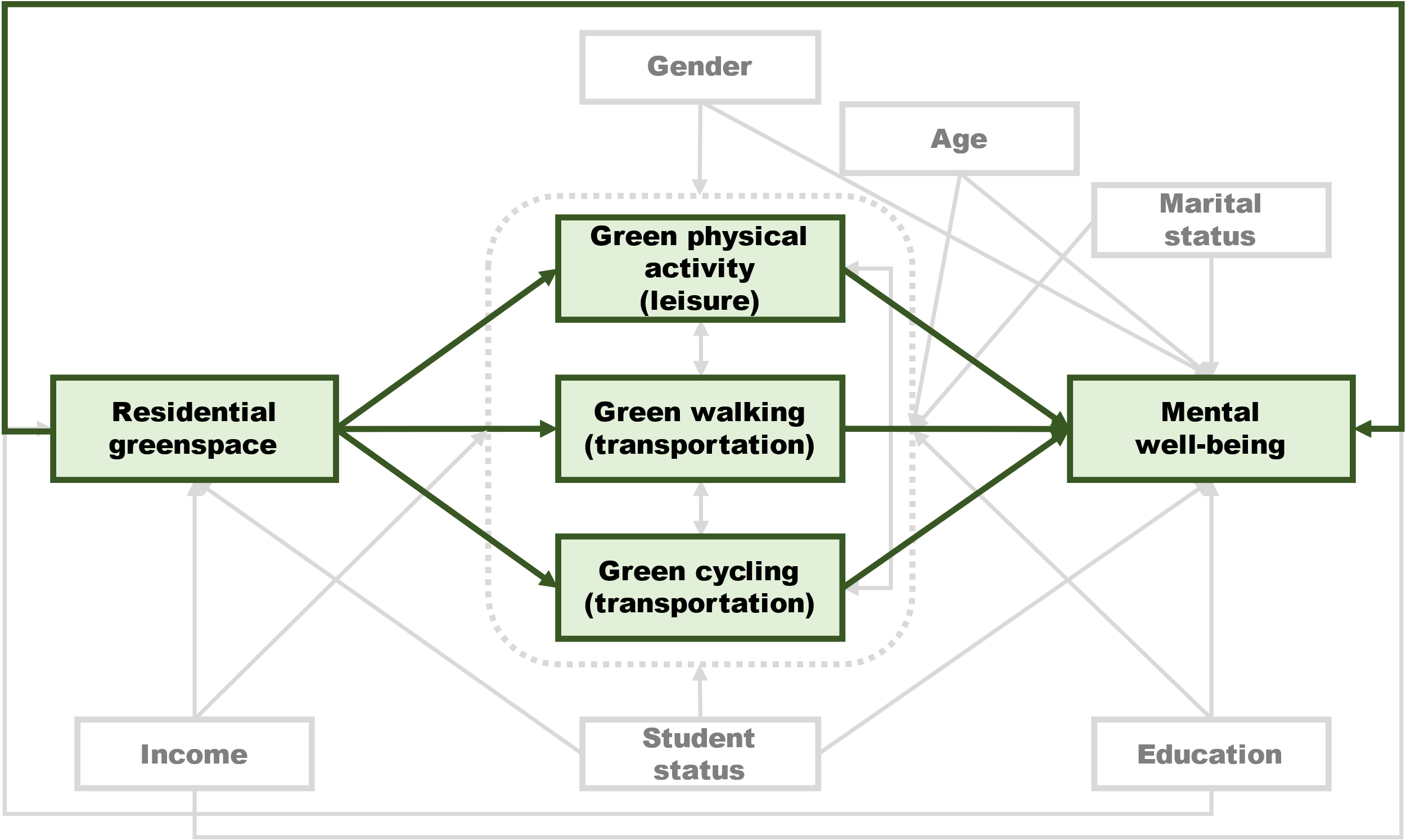


**Supplementary Figure 1.** The conceptual model. Green lines indicate pathways between variables of interest, and grey lines indicate control pathways.

**
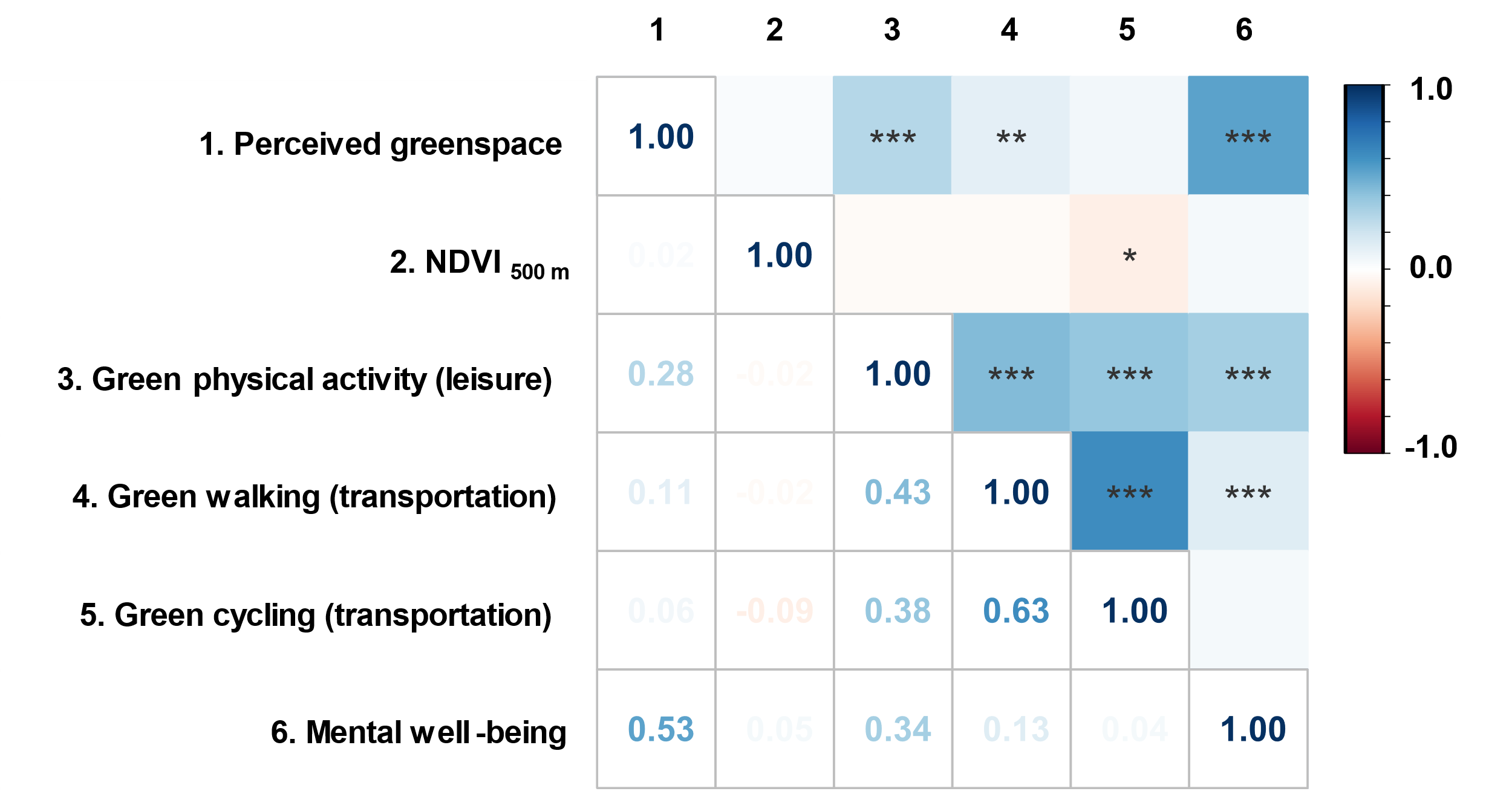
**

**Supplementary Figure 2.** The spearman’s correlation. Number in cells indicate Spearman’s rho; *, *p* < 0.05; **, *p* < 0.01; ***, *p* < 0.001.

**Supplementary Table 1.** Participants’ characteristics

| Variable | Category | Percentage | N |
| --- | --- | --- | --- |
| Gender | Male | 78.7% | 526 |
|  | Female | 21.3% | 142 |
| Age | ≤15 year | 0.1% | 1 |
|  | 15-25 | 41.0% | 274 |
|  | 26-35 | 46.0% | 307 |
|  | 36-45 | 11.2% | 75 |
|  | 45-55 | 1.2% | 8 |
|  | 56-65 | 0.4% | 3 |
|  | 66-75 | 0.1% | 1 |
|  | >75 year | 0.0% | 0 |
| Household income | 0-5000 RMB | 5.4% | 36 |
|  | 5001-10000 | 31.4% | 210 |
|  | 10001-15000 | 28.9% | 193 |
|  | 15001-20000 | 20.7% | 138 |
|  | 20001-25000 | 8.5% | 57 |
|  | >25000 RMB | 5.1% | 34 |
| Education | Lower than Bachelor degree | 24.3% | 162 |
|  | Bachelor or equivalent degree | 63.8% | 426 |
|  | Higher than Bachelor degree | 12.0% | 80 |
| Student status | Student | 47.9% | 320 |
|  | Non-student | 52.1% | 348 |
| Marital status | Married | 39.7% | 265 |
|  | Unmarried | 60.3% | 403 |
